# Multiple Myeloma With Amplification of Chr1q: Therapeutic Opportunity and Challenges

**DOI:** 10.3389/fonc.2022.961421

**Published:** 2022-07-14

**Authors:** Romanos Sklavenitis-Pistofidis, Gad Getz, Irene Ghobrial, Maria Papaioannou

**Affiliations:** ^1^ Harvard Medical School, Boston, MA, United States; ^2^ Department of Medical Oncology, Dana-Farber Cancer Institute, Boston, MA, United States; ^3^ Broad Institute of Massachusetts Institute of Technology (MIT) and Harvard, Cambridge, MA, United States; ^4^ Medical School, Aristotle University of Thessaloniki, Thessaloniki, Greece; ^5^ Department of Pathology, Massachusetts General Hospital, Boston, MA, United States; ^6^ Hematology Unit, 1st Internal Medicine Department, AHEPA University Hospital, Thessaloniki, Greece

**Keywords:** multiple myeloma, chr1q, amplification, precision medicine, therapy

## Abstract

Multiple myeloma (MM) is an incurable plasma cell malignancy with a heterogeneous genetic background. Each MM subtype may have its own therapeutic vulnerabilities, and tailored therapy could improve outcomes. However, the cumulative frequency of druggable targets across patients is very low, which has precluded the widespread adoption of precision therapy for patients with MM. Amplification of the long arm of chromosome 1 (Amp1q) is one of the most frequent genetic alterations observed in patients with MM, and its presence predicts inferior outcomes in the era of proteasome inhibitors and immunomodulatory agents. Therefore, establishing precision medicine for MM patients with Amp1q stands to benefit a large portion of patients who are otherwise at higher risk of relapse. In this article, we review the prevalence and clinical significance of Amp1q in patients with MM, its pathogenesis and therapeutic vulnerabilities, and discuss the opportunities and challenges for Amp1q-targeted therapy.

## Background

Multiple myeloma (MM) is an incurable plasma cell malignancy that is usually confined to the bone marrow (1). It is preceded by two asymptomatic conditions, monoclonal gammopathy of undetermined significance (MGUS) and smoldering multiple myeloma (SMM) (2). These are delineated by the amount of tumor detected in the bone marrow or monoclonal protein detected in the blood, and thus have a different risk of progression to overt disease (2). Approximately 5% of people over the age of 40, and 17% of Black people over the age of 50 have MGUS (3, 4), but on average only 1% of those will develop MM every year (5). As such, despite the high prevalence of gammopathy in the general population, MM accounts for 1% of all cancers (1). Approximately 0.5% of people over the age of 40 years have SMM (6); their progression risk follows a logistic growth model with a yearly risk of 10% for the first 5 years and a gradual decrease in risk after that until an MGUS-like slope is reached (7). The mixed nature of this risk pattern indicates that, while useful, the clinical definitions of MGUS and SMM are broad and encompass multiple biological entities (8–13). Likewise, the clinical entity of MM is, in biological terms, a collection of different entities with similar cell morphology and clinical presentation (14). Cytogenetics-based and gene expression-based classifications of MM have been validated and delineate several subtypes of disease with variable outcomes (15–19). Since their underlying biology is different, it is reasonable to assume that each subtype may have its own therapeutic vulnerabilities and that tailored therapy or precision medicine may improve patient outcomes (20, 21). In practice, though, today the only subtype with an actionable, class-wide vulnerability is MM with translocation t(11;14), which is sensitive to BCL2 inhibition (20, 22, 23). Arguably, MM with t(11;14) is also the subtype with the most distinctive biological characteristics, including lymphoplasmacytic morphology, frequent expression of B-cell surface antigens, and a possible origin in bone marrow pro-B cells, as opposed to most other myelomas, which arise from post-germinal center B cells (24–26). Although the survival of patients with MM has improved significantly over the past decades (27), that has been thanks to effective one-size-fits-all regimens that are tailored to the cell type rather than the genetic alterations of the tumor (28). The limited success of precision medicine in MM is partly because class-defining alterations are either translocations that dysregulate undruggable targets, such as *WHSC1* or *MAF*, or somatic copy number alterations (SCNAs), whose vulnerabilities are challenging to study and target. It is also due to the low cumulative frequency of actionable mutations, which precludes a broader adoption of precision therapy for patients with MM (20, 29, 30).

The amplification of the long arm of chromosome 1 (chr1q) is one of the most common SCNAs in patients with MM (31). Its presence is associated with poor outcomes (32). It follows that a large fraction of patients with MM stand to benefit from precision therapy approaches for MM with amplification of chr1q (Amp1q). In this review article, we summarize the evidence surrounding the prevalence and clinical significance of Amp1q in MM and its precursors, pathogenesis, and therapeutic vulnerabilities and discuss the opportunities and challenges in establishing precision medicine for MM patients with Amp1q.

## Prevalence of Amp1q in patients with MM and its precursors

**Figure 1:**
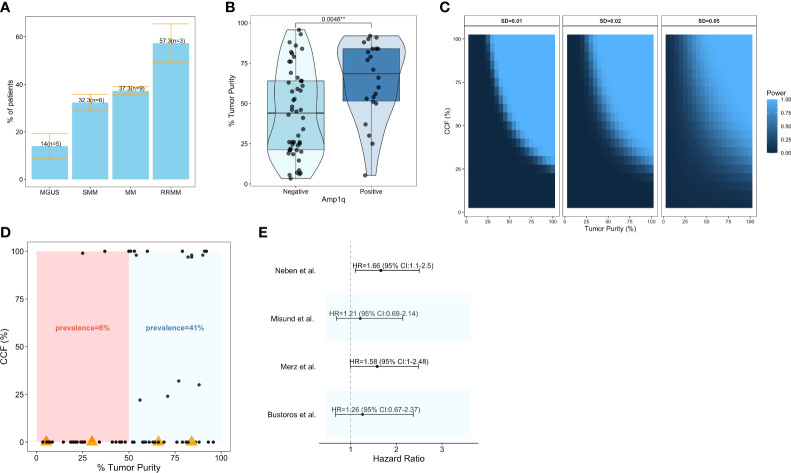
Prevalence and clinical significance of Amp1q in patients with MM and its precursors. **(A)** Barplots visualizing the mean ± standard error of the prevalence of Amp1q per disease stage. The number of studies (n) considered in those estimates is also visualized. **(B)** Boxplots comparing the tumor purity of samples in which Amp1q was detected to those in which it was not detected in Bustoros et al. (8). **(C)** Power analysis for the detection of arm-level copy number variants in sequencing experiments as a function of tumor purity (x-axis) and the abnormality’s cancer cell fraction (CCF). Each panel corresponds to a different standard deviation (SD). **(D)** Scatterplot of tumor purity (x-axis) and CCF (y-axis) in patients from Bustoros et al. (8). Samples in which Amp1q was not detected by whole-exome sequencing are shown with a CCF of 0. Samples in which Amp1q was not detected by whole-exome sequencing but was detected by fluorescence *in situ* hybridization (FISH) are shown with an orange triangle. **(E)** Forest plot summarizing the impact of Amp1q on the risk of progression from smoldering multiple myeloma to overt disease in four studies. Its effect is visualized as a hazard ratio with a 95% confidence interval.

Increases in the copy number of chr1q are sometimes categorized as “gains” when the total number of copies is 3 (i.e., 1 extra copy), and “amplifications” when the total number of copies is larger (i.e., 2 or more extra copies) (33). In this review, the term “amplification” will be used to denote all increases in copy number, irrespective of the number of copies gained, as not all publications summarized here present data separately for gains and amplifications; it should be noted, however, that amplifications may confer more aggressive disease compared to gains (32, 33). The amplification of chr1q is the second most common arm-level SCNA in patients with MM, following Del13q (31). Patients with newly diagnosed MM have a high frequency of Amp1q by fluorescence *in situ* hybridization (FISH) [43%, n = 479, 43% of whom had 4 copies or more (34); 40.5%, n = 205, 22% of whom had 4 copies or more (35); 39%, n = 767 (36); 37%, n = 880, 25% of whom had 4 copies or more (37); 33%, n = 520 (38)], comparative genomic hybridization (CGH) [45%, n = 51 (39)], or Multiplex Ligation-dependent Probe Amplification (MLPA) [34%, n = 1,716 (37)]. In a whole-exome sequencing (WES) study of 1,074 patients with newly diagnosed MM, 29% had Amp1q, and 21% of those had 4 copies or more (29); whereas, in a whole-genome sequencing (WGS) study of 871 patients, 35% had Amp1q (40). Similar prevalence estimates have been reported in patients with SMM either by FISH [45%, n = 31 (34); 41%, n = 114 (41); 30%, n = 245 (42)] or next-generation sequencing (NGS) [25%, n = 77 (11); 28.5%, n = 214 (8); 24.4%, n = 90 (10)]. This is consistent with exome- or genome-wide analyses showing similar genomic profiles in patients with SMM and newly diagnosed MM (8, 10–13). However, in patients with MGUS, the prevalence of Amp1q has been reported to be lower by FISH [0%, n = 14 (34); 20%, n = 88 (37); 29%, n = 79 (43); 16.8%, n = 155 (44)] and NGS [4.3%, n = 23 (10)]; this is consistent with reports of lower prevalence for other secondary genomic alterations in MGUS, such as Del13q and Del17p (13, 43, 44). Lastly, the prevalence of Amp1q in patients with relapsed/refractory MM has been reported to be higher by FISH [72%, n = 45, 60% of whom had 4 copies or more (34); 44%, n = 81 (45); ~56%, n = 178 (46)]. Across all studies discussed here, Amp1q is present on average in approximately 14% (SE: 5.3%) of patients with MGUS, 32.3% of patients with SMM (SE: 3.5%), 37.3% of patients with MM (SE: 1.7%), and 57.3% (SE: 8.1%) of patients with RRMM (**Figure 1A**). Its increasing frequency along the stages of disease progression suggests a potential role for Amp1q in disease aggressiveness.

It is important to note that the true prevalence of Amp1q may be underestimated in early-stage disease where the tumor purity of the samples (i.e., the fraction of malignant cells in the sample) can be low, and secondary alterations like Amp1q are often subclonal with low/intermediate cancer cell fraction (CCF) (i.e., the fraction of malignant cells with the abnormality). In Bustoros et al. (8), a study of WES in patients with SMM, the average tumor purity was 47%, and Amp1q was detected in 28% (n = 214) of patients; indeed, the detection of Amp1q was significantly associated with higher tumor purity (Wilcoxon, p = 0.004), suggesting that the true frequency of this abnormality may be higher in this cohort (**Figure 1B**). To estimate our power to detect Amp1q in a sequencing experiment of good quality, we can assume that chr1q copy ratios are normally distributed with relatively small standard deviations (SD) and that they are a function of purity and ploidy as outlined in Carter et al. (47). By requiring a copy ratio of at least 1.1 to detect the abnormality, then in a sample with 45% tumor purity and SD of 0.01 we would be well powered (power >0.8) to detect Amp1q provided its CCF is at least 65% (**Figure 1C**), which we argue may not be the case in patients with early-stage disease. Indeed, in Bustoros et al. (8), the majority of samples in which Amp1q was detected had a tumor purity of at least 50%, and if we calculated the prevalence of Amp1q based on samples with at least 50% tumor purity, it would be closer to that reported by FISH (41%) (**Figure 1D**). Furthermore, different studies may have different amounts of noise in the data, necessitating different thresholds for calling the abnormality in a sample. This problem is not unique to NGS studies. For example, when FISH is used to detect Amp1q, a CCF threshold is used to report the detection of the abnormality, which is usually around 10–30% (i.e., the abnormality needs to be present in at least this fraction of cancer cells studied for it to be called), and which can differ from study to study. Other sources of this bias in FISH studies include the type of cells tested (e.g., mononuclear cells or magnetically sorted CD138+ plasma cells), the number of nuclei tested (typically 100–200 nuclei), and whether cytoplasmic kappa/lambda staining is performed to enrich for cells expressing the light chain of the clone, all of which can affect the probability of detecting a subclonal Amp1q (48). This is important to keep in mind as we consider estimates of the clinical significance of Amp1q since different studies may have different proportions of patients with low-CCF Amp1q subclones, which may impact survival as much as clonal abnormalities (49).

## Clinical significance of Amp1q in patients with MM and its precursors

In patients with SMM, the detection of Amp1q by FISH conferred a ~60% higher risk of progression to overt MM in two different studies (42, 50). However, Amp1q was not a significant predictor of progression in two other studies that used WES and custom panels to detect it (**Figure 1E**) (8, 10). While multiple studies have shown an excellent correlation between FISH and NGS in patients with MM (51–53), it is possible that FISH (which studies single nuclei) can detect subclonal Amp1q in more patients than NGS (which studies DNA fragments in bulk). This could explain the dampening of the survival impact of Amp1q in NGS studies of patients with SMM. In Bustoros et al. (8), FISH detected Amp1q in four more patients than WES, and for two of those, the tumor purity was above 65%, suggesting that the abnormalities missed were subclonal (**Figure 1D**). Nevertheless, including these 4 patients in the Amp1q group did not change its effect on the risk of progression, suggesting that while detection sensitivity is an issue, it does not fully explain the differences observed in hazard ratios.

As patients with newly diagnosed MM receive treatment, the effect of Amp1q detection on their overall survival (OS) is also a function of the type of treatment administered, and therefore can change as the standard of care improves. In patients with newly diagnosed MM who were treated with vincristine, adriamycin, and dexamethasone (VAD) followed by melphalan and autologous stem cell transplantation (ASCT) in the IFM 99-02, IFM 99-04, and CMG2002 trials, the detection of Amp1q by FISH or SNP array was associated with significantly shorter OS in multivariate analyses (38, 54, 55). Furthermore, the detection of Amp1q was associated with significantly shorter OS in patients with newly diagnosed MM who were treated on the Total Therapy 2 (TT2), Myeloma IX, and the GMMG-HD-3/HD-4 trials, which started incorporating thalidomide and bortezomib into chemotherapy induction regimens (34, 56, 57). However, except in the TT2 study, Amp1q was not a significant predictor in the multivariate setting, raising questions about its usefulness in risk models, given that it frequently co-occurs with other high-risk abnormalities (33, 34, 56, 57). This was also the case in an analysis that looked at patients from the Mayo Clinic who were treated with high-dose therapy and transplantation, as well as an analysis of a subset of patients from the TT2 trial (58). Nevertheless, recent data from patients who were treated with proteasome inhibitors and immunomodulatory agents indicate that Amp1q is a significant independent risk factor for patients with newly diagnosed MM in this era (31, 59–64).

In summary, in the era of proteasome inhibition and immunomodulatory agents, Amp1q is a significant predictor of poor outcomes in patients with newly diagnosed MM. In patients with SMM, Amp1q may be a risk factor for progression, although more and larger studies are needed to confirm this and prove that its effect is independent of other high-risk abnormalities, such as Myc translocations. Therefore, establishing precision therapy for patients with MM and Amp1q stands to benefit a large fraction of patients who are still at higher risk of poor outcomes, despite improvements in plasma cell-targeting therapies.

## Pathogenesis of MM with Amp1q

Chromosome 1q can be amplified as part of a trisomy (where an extra copy of the entire chr1 is generated, perhaps due to missegregation during mitosis), or whole-genome doubling, but arm-level events (Amp1q) are observed in more than 75% of patients (65). Patients with MM and Amp1q show a preponderance of breakpoints in the pericentromeric heterochromatin region of cytoband 1q12 (66). Chromosome 1 has the longest pericentromeric heterochromatin region, which is rich in satellite 2 and 3 DNA repeats and is one of the most frequent breakpoint locations in cancer (67, 68). The pericentromeric chromatin of Chromosome 1 is maintained in a repressed state (heterochromatin) through epigenetic regulation, involving the tri-methylation of the 9th lysine residue (H3K9m3) of histone 3 by Suv39h methyltransferases and the subsequent binding of these methylated residues by heterochromatin protein 1 (HP1) (67, 69, 70). Suv39h methyltransferases accumulate around centromeres during mitosis, and together with HP1, orchestrate the methylation of pericentromeric satellite repeats by DNA methyltransferases 3A and 3B (*DNMT3A*, *DNMT3B*) (71, 72). These interactions play a key role in chromosome segregation and stability by preserving pericentromeric chromatin in its more condensed form. Loss of Suv39h leads to hypomethylation of the pericentromeric heterochromatin, chromosomal instability, and malignant transformation *in vivo* (70). Germline mutations in *DNMT3B* in patients with immunodeficiency, centromeric instability, and facial anomalies (ICF) syndrome lead to hypomethylation and decondensation of pericentromeric heterochromatin in chr1; this results in elongated, thread-like pericentromeric chromatin and multiradial chromosomal formations comprising multiple copies of whole arms, which lead to missegregation, chromosomal translocations, and copy number gains in chr1 (73, 74). Similar chromosomal anomalies can be induced in human lymphocytes with drug-induced pericentromeric hypomethylation, using, for example, the methyltransferase inhibitor 5-azacytidine (75–77).

**Figure 2:**
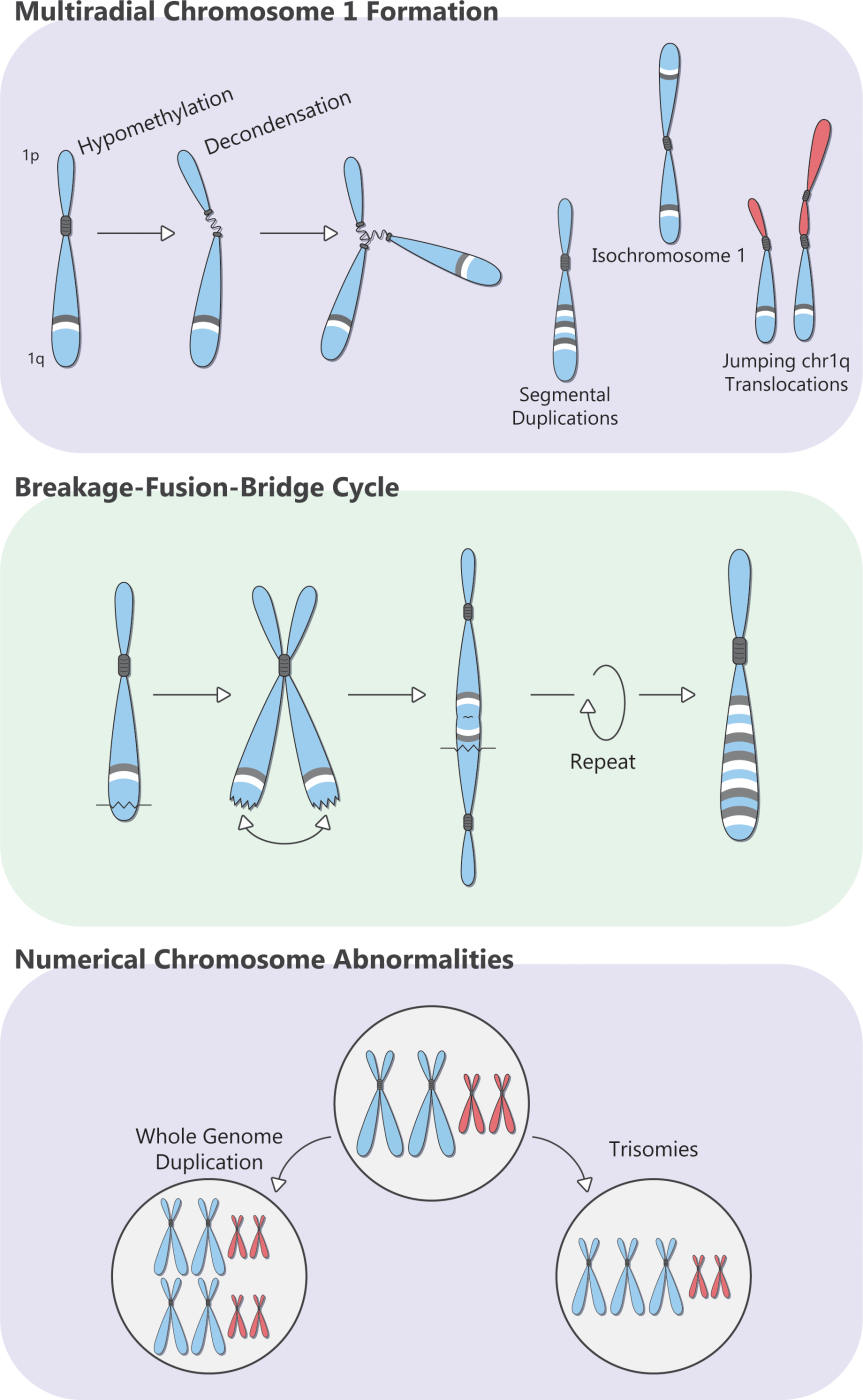
Overview of the pathogenesis of Amp1q in patients with MM.

Multiple myeloma is characterized by global hypomethylation with significant interpatient variability, although MM with Amp1q does not appear to have a characteristic methylation trace compared with other cytogenetic abnormalities (78, 79). In patients with MM, metaphase cytogenetics shows active decondensation of pericentromeric heterochromatin with multiradial chr1 formations, which leads to jumping chr1q translocations (whereby the same donor fragment is translocated onto two or more chromosomes, in different cells), isochromosome 1q (whereby chr1p is deleted, and chr1q is duplicated), segmental duplications (whereby certain segments of chr1q are duplicated, sometimes repeatedly so), and breakage–fusion–bridge cycles (**Figure 2**) (80–83). Importantly, these alterations can continue to evolve, producing subclones with more copies of chr1q and more complex karyotypes. While such abnormalities can be reproduced *in vitro* by treating peripheral blood mononuclear cells from patients with MM with the hypomethylating agent 5-azacytidine (77), no drug has, thus far, been able to reverse or halt this state of hypomethylation and chromosomal instability in MM cells. More studies on the pathogenesis of Amp1q in patients with MM may reveal novel class-wide vulnerabilities and improve patient outcomes.

## Therapeutic vulnerabilities of MM with Amp1q

**Figure 3:**
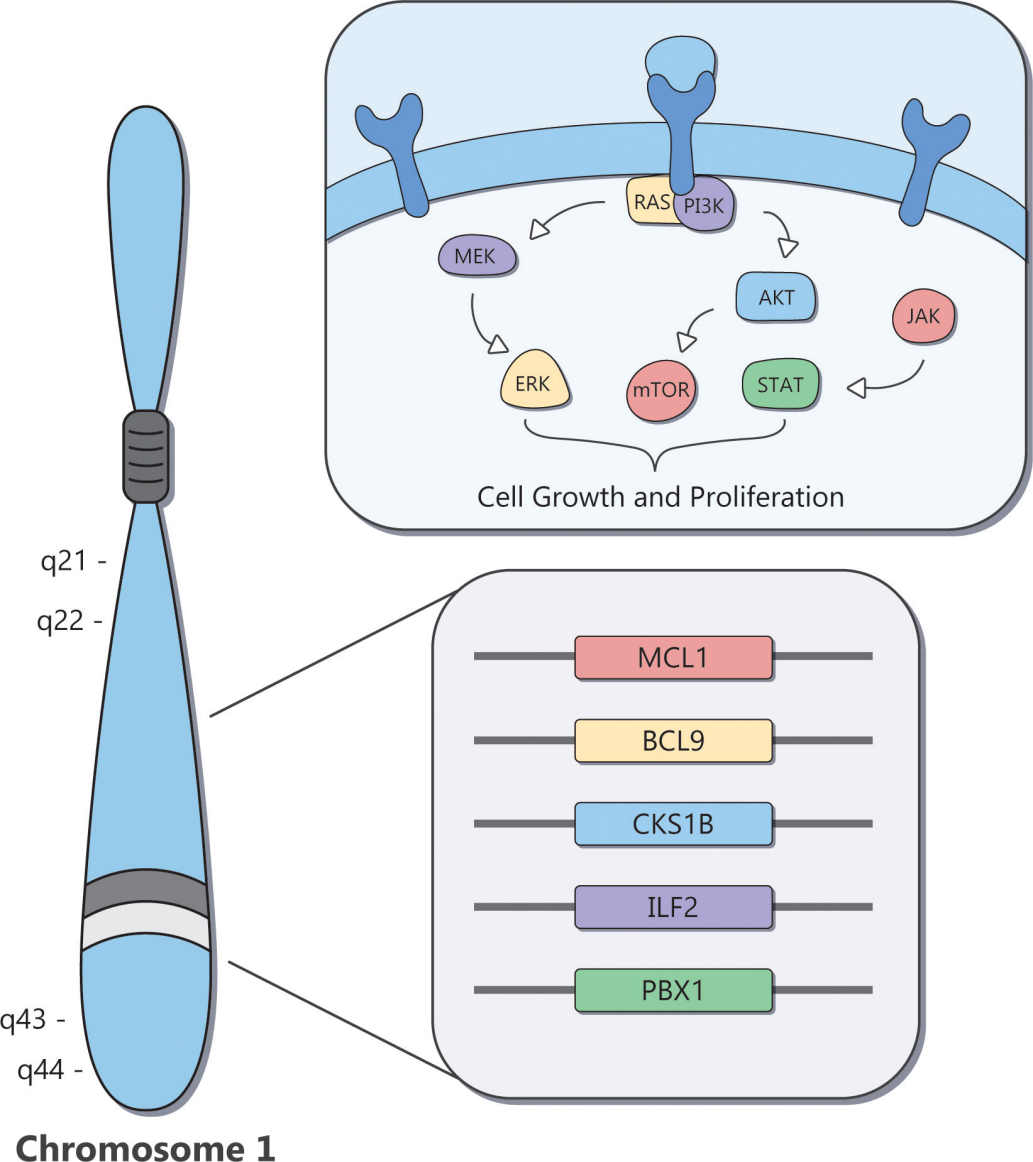
Overview of the therapeutic vulnerabilities of MM with Amp1q.

The identification of therapeutic vulnerabilities for MM with Amp1q has focused on genes located in cytoband 1q21 (**Figure 3**). Despite Amp1q typically being an arm-level abnormality, minimal common regions have been identified in cytobands 1q21–1q22 and 1q43–1q44 (84). Such regions are presumed to contain genes critical for tumor cell proliferation and survival; in this case, genes such as *MCL1*, *BCL9*, *CKS1B*, and *ILF2*, and members of the ubiquitin-proteasome pathway are found in the affected regions (84). CDC28 Protein Kinase Regulatory Subunit 1B (*CKS1B*) promotes the ubiquitination and degradation of p27, a cell cycle inhibitor encoded by *CDKN1B* (85, 86), and activates STAT3 and the MEK/ERK pathway (87). Inhibition of STAT3 and MEK1, or inhibition of p27 degradation *via* a NEDD8 inhibitor, may be particularly effective in tumors with high levels of CKS1B, such as MM with Amp1q (86, 88). Myeloid Cell Factor 1 (*MCL1*) encodes an antiapoptotic protein that most MM tumors depend on for survival through both tumor-intrinsic and extrinsic mechanisms (89–94). Multiple myeloma with Amp1q is particularly dependent on MCL1, which results in pronounced sensitivity to MCL1 inhibitors (95–97). B-cell lymphoma 9 (*BCL9*) acts as a co-activator of β-catenin, and promotes cell proliferation, metastasis, and angiogenesis in MM (98). Disruption of its protein–protein interaction with β-catenin, which may be particularly relevant for MM with Amp1q, can suppress tumor growth *in vivo*. However, therapeutically targeting this signaling pathway has proved challenging (99, 100). Interleukin Enhancer Binding Factor 2 (*ILF2*) encodes NF45, a subunit of the transcription factor NFAT, and regulates genomic stability in MM with Amp1q; knockdown of *ILF2 in vivo* leads to significantly prolonged OS, although currently no drug has been shown to degrade this transcription factor in patients with MM and Amp1q (101). In recent years, technological advances have enabled the systematic interrogation of all of chr1q using large-scale screens and multi-omics approaches, which have nominated new actionable targets, including the PI3K pathway (96), the kinase CLK2 (102), and the transcription factor PBX1 (103).

In summary, the presence of Amp1q in a MM tumor confers specific therapeutic vulnerabilities, some of which may already be actionable today. However, these discoveries have not been translated into clinical practice, as candidate drugs have not been tested in patients with Amp1q yet and have been hampered by suboptimal efficacy and toxicity profiles. Furthermore, it is unclear how these vulnerabilities may be modified by the presence of other genomic abnormalities and whether further patient stratification will be necessary to achieve optimal responses to targeted therapy.

## Opportunity and challenges in targeting MM with Amp1q

Because of its frequency and its impact on patient outcomes, multiple myeloma with Amp1q represents an opportunity for precision medicine to improve the lives of many patients who have not benefitted as much from the therapeutic developments of recent years. Furthermore, the discovery of vulnerabilities that are either specific to Amp1q or enhanced by the presence of Amp1q indicates that precision approaches can indeed be developed for patients with this abnormality. A major limitation in developing precision medicine for MM with Amp1q is the lack of genome engineering methods that enable the generation of arm-level amplifications in human cell line models. Methods exist for the generation of single-chromosome trisomies in human cells. However, those are not frequently seen in patients with MM and Amp1q, possibly due to the existence of potent tumor suppressors on chr1p, which is frequently deleted in patients with MM (104–106). In the absence of syngeneic models of MM with Amp1q, we are forced to draw conclusions from suboptimal comparisons of cell lines that have Amp1q to cell lines that do not, which differ by more than just the presence or absence of Amp1q. Moreover, approximately 91% of MM cell lines have Amp1q, which restricts the pool of negative control cell lines available (34). It is also unclear whether all amplifications of chr1q generate similar vulnerabilities; perhaps certain targets are dose-dependent (i.e., a certain number of chr1q copies is required), or depend upon the type of the amplification (e.g., segmental duplication, translocation, isochromosome), the borders of the amplification (i.e., is the target contained within the borders or not), the size of the amplification (e.g., focal or arm-level), or the co-occurrence of Amp1q with another genomic abnormality, which may modify its effect on cell fitness. Furthermore, many of the dependencies discovered may not be directly actionable (84, 96). Considering the vulnerabilities that are actionable today, none have been tested in patients with Amp1q yet. MEK inhibitors may be effective in combination with BCL2 and/or PD-L1 inhibitors in patients with MM (20), but PI3K inhibitors can be quite toxic and often lead to lackluster responses (20, 107), and MCL1 inhibitors may be effective but may not be tolerated well, generating concerns of cardiotoxicity, for example (20, 108). Furthermore, resistance mechanisms have been described, including MEK/ERK activation following PI3K inhibition and vice versa (109), and BCL2 or BCL_XL_ activation following MCL1 inhibition (95, 110), suggesting that combinatorial regimens may be necessary for successful treatment of these tumors.

## Conclusion

The survival of patients with MM has improved drastically over the last couple of decades. However, patients with high-risk genomic features, such as Amp1q, have not benefited as much. The variety of methods used to detect Amp1q complicates the interpretation of changes in its prevalence over stages of disease progression and its clinical significance in newly diagnosed patients. However, multiple studies confirm that its prevalence is high and that it is an independent predictor of poor outcomes in multivariate analyses even in the era of proteasome inhibitors and immunomodulatory agents. In patients with SMM, larger studies are needed to assess the clinical significance of Amp1q in analyses that account for other genomic and/or clinical variables. Tailoring treatment to the underlying abnormality could improve outcomes for patients with MM and Amp1q. However, developing precision approaches has proved challenging. Despite the role of hypomethylation in the generation and continuous evolution of Amp1q, no epigenetic therapeutic approaches have been developed to target it. Instead, precision approaches for MM with Amp1q typically target genes that are located on 1q21, the most commonly amplified region of chr1q, such as *MCL1*. When actionable, these approaches may be effective. However, they are often toxic and may require a combination with other inhibitors to preempt escape mechanisms and drug resistance. Until now, none of these approaches has been specifically tested in patients with Amp1q, where the therapeutic window may be different, and it is unclear how the presence of other abnormalities may modify the effect of these treatments. Establishing precision therapy for patients with MM and Amp1q will require clinical trials to systematically enroll patients with Amp1q and report results for this subgroup of patients.

## Author Contributions

RS-P conducted the literature review and analysis, and wrote the manuscript. GG, MP, and IG reviewed, edited and approved the manuscript. All authors contributed to the article and approved the submitted version.

## Funding

The authors would like to thank Anna V. Justis, PhD, for her editing services, Sarah Nersersian, MSc, for illustration support, and Nicholas J. Haradhvala, PhD, for his suggestions regarding the power analysis for the detection of SCNAs in sequencing experiments. RS-P is supported by the Multiple Myeloma Research Foundation Fellowship Award, the International Waldenstrom’s Macroglobulinemia Foundation’s Robert A. Kyle Award, and the Claudia Adams-Barr Award for Innovative Basic Cancer Research. GG is partially supported by the Paul C. Zamecnik Chair in Oncology at Massachusetts General Hospital Cancer Center. IMG and GG would like to acknowledge funding from the Dr. Miriam and Sheldon G. Adelson Medical Research Foundation and the Multiple Myeloma Research Foundation (MMRF) and Dana-Farber Prevention Program. This research was also supported by a Stand Up To Cancer Dream Team Research Grant (Grant Number: SU2C-AACR-DT-28-18). Stand Up To Cancer is a program of the Entertainment Industry Foundation. Research grants are administered by the American Association for Cancer Research, the scientific partner of Stand Up To Cancer. Opinions, interpretations, conclusions, and recommendations are those of the author(s) and are not necessarily endorsed by Stand Up To Cancer, the Entertainment Industry Foundation, or the American Association for Cancer Research.

## Conflict of Interest

GG receives research funds from IBM and Pharmacyclics, and is also an inventor on patent applications filed by the Broad Institute related to MSMuTect, MSMutSig, POLYSOLVER, SignatureAnalyzer-GPU, and MSIDetect. He is also a founder, consultant, and holds privately held equity in Scorpion Therapeutics. IG has a consulting or advisory role with AbbVie, Adaptive, Amgen, Aptitude Health, Bristol Myers Squibb, GlaxoSmithKline, Huron Consulting, Janssen, Menarini Silicon Biosystems, Oncopeptides, Pfizer, Sanofi, Sognef, Takeda, The Binding Site, and Window Therapeutics; has received speaker fees from Vor Biopharma and Veeva Systems, Inc.; and her spouse is the CMO and equity holder of Disc Medicine.

The remaining authors declare that the research was conducted in the absence of any commercial or financial relationships that could be construed as a potential conflict of interest.

## Publisher’s Note

All claims expressed in this article are solely those of the authors and do not necessarily represent those of their affiliated organizations, or those of the publisher, the editors and the reviewers. Any product that may be evaluated in this article, or claim that may be made by its manufacturer, is not guaranteed or endorsed by the publisher.
